# Supplementary material for: Clinical Characteristics in Early Childhood Associated with a Nevus-Prone Phenotype in Adults from Tropical Australia: Two Decades of Follow-Up of the Townsville Preschool Cohort Study
Source: Int J Environ Res Public Health. 2020 Nov 23;17(22):8680. doi: 10.3390/ijerph17228680 (PMC7700251; doi:10.3390/ijerph17228680)
Supplement: Supplementary file 1 [file ijerph-17-08680-s001.pdf]

Table S1. Baseline characteristics of a sample of 51 cohort participants still residing in Townsville, Queensland who were re-examined for melanocytic nevi (MN) in 2016 compared to the original Townsville Preschool cohort from which they were drawn.

Characteristic	Original Cohort examined twice for MN as children [n = 484] n (%)	Sample of cohort residing in Townsville who were examined for MN as children & in 2016 as adults [n = 51] n (%)
**Gender**		
Female	242 (50%)	28 (54.9%)
Male	242 (50%)	23 (45.1%)
**Age at baseline (years)**		
1	95 (19.6%)	10 (19.6%)
2	92 (19.0%)	8 (15.7%)
3	92 (19.0%)	14 (27.5%)
4	77 (15.9%)	8 (15.7%)
5	75 (15.5%)	5 (9.8%)
6	53 (11.0%)	6 (11.8%)
**Highest Maternal Education**		
Some High School	131 (27.1%)	11 (21.6%)
Completed Senior Schooling	242 (50.0%)	25 (49.0%)
Tertiary Education	111 (22.9%)	15 (29.4%)
**Eye color as child (baseline)**		
Brown	109 (22.5%)	15 (29.4%)
Hazel	110 (22.7%)	11 (21.6%)
Blue/green/grey	265 (54.8%)	25 (49.0%)
**Hair color as child (baseline)**		
Brown	147 (30.4%)	19 (37.3%)
Blond/fair	321 (66.3%)	32 (62.7%)
Red	16 (3.3%)	0 (0%)
**Natural skin color (baseline)**		
Olive	1 (0.2%)	1 (2.0%)
Medium	104 (21.5%)	9 (17.6%)
Fair	379 (78.3%)	41 (80.4%)
**Freckling as a child (baseline)**		
Absent	236 (48.8%)	23 (45.1%)
Present	248(51.2%)	28 (54.9%)
**Solar lentigines on shoulders as a child (baseline)**		
Absent	474 (97.9%)	49 (96.1%)
Present	10 (2.1%)	2 (3.9%)
**Tanning ability as a child (baseline)[1]**		

Very brown & deeply tanned	44 (9.2%)	5 (10.0%)
Moderately tanned	289 (60.3%)	31 (62.0%)
Slightly tanned	52 (10.9%)	7 (14%)
Not suntanned at all	94 (19.6%)	7 (14%)
**MN count at baseline**		
Mean ± SD	40.5 ± 34.0	38.3 ± 34.7
Median [IQR]	34 [13.25, 58.75]	32 [13, 55]
**MN 5 + mm as a child (baseline)**		
Absent	422 (87.2%)	44 (86.3%)
Present	62 (12.8%)	7 (13.7%)
**MN 3 + mm as a child (baseline)**		
Absent	225 (46.5%)	24 (47.1%)
Present	259 (52.9%)	27 (52.9%)
**Sunburnt before 7 years-old (baseline)**		
No	189 (39.0%)	20 (39.2)
Yes	295 (61.0%)	31 (60.8)

1 missing questionnaire data for n = 5 participants in the original cohort and n = 1 participant in the 2016 follow-up sample.

Table S2. Distribution of incident and prevalent melanocytic nevi (MN) shown by age at follow-up in 2016.

Age	n (%)	Incident MN Count	Total MN Count (Prevalent MN)	MN ≥2mm (Prevalent MN)	MN ≥5mm (Large Prevalent MN)
**21 years**	5 (9.8)				
Mean ± SD (Range)		155.8 ± 86.7 (40-258)	178.8 ± 88.1 (46-274)	108 ± 72 (13-209)	1.8 ± 1.6 (0-4)
Median [IQR]		148 [76, 239.5]	169 [104.5, 258]	113 [44.5, 169]	1 [0.5, 3.5]
**25 years**	5 (9.8)				
Mean ± SD (Range)		227.6 ± 65.9 (181-342)	232.2 ± 70.1 (184-354)	70.4 ± 42.3 (33-127)	2.8 ± 4.7 (0-11)
Median [IQR]		214 [183, 279]	216 [185, 287.5]	48 [36.5, 115.5]	1 [0, 6.5]
**26 years**	6 (11.8)				
Mean ± SD (Range)		136.8 ± 89.0 (54-240)	232.2 ± 70.1 (184-354)	27.8 ± 19.2(4-56)	2.2 ± 3.5(0-9)
Median [IQR]		111 [54.25, 240]	125 [59.75, 253.25]	29.5 [7.75, 43.25]	0.5 [0, 4.5]
**27 years**	10 (19.6)				
Mean ± SD (Range)		182.7 ± 98.3 (40-331)	204.4 ± 104.5 (46-347)	62.4 ± 59.7 (11-222)	4.5 ± 6.9 (0-21)
Median [IQR]		172 [105.25, 268]	205 [118.25, 291.5]	45.5 [30.5, 72]	1 [0, 9.25]
**28 years**	9 (17.6)				
Mean ± SD (Range)		362.2 ± 192.8 (106-706)	410.7 ± 206.3 (141-753)	127 ± 76.9 (37-259)	2 ± 2.1 (0-5)
Median [IQR]		424 [188, 477]	479 [207, 544.5]	112 [62.5, 203]	2 [0, 4]
**29 years**	6 (11.8)				
Mean ± SD (Range)		279.2 ± 242.6 (86-668)	350.7 ± 291.5 (122-849)	145.5 ± 167.1 (32-481)	7.2 ± 14.2 (0-12)

Median [IQR]	164 [95.75, 538.25]	201 [156.5, 635.25]	87 [55.25, 212.5]	1.5 [0, 12]
**≥30 years** 10 (19.6)				
Mean ± SD (Range)	227 ± 172.2 (63-565)	293.6 ± 192.8 (98-686)	61.7 ± 43.4 (21-176)	1.4 ± 1.9 (0-6)
Median [IQR]	159 [85.8, 351.5]	227 [135.25, 412.75]	49.5 [39.25, 68.5]	1 [0, 2.25]
**Total** **51 (100)**				
Mean ± SD (Range)	230.8 ± 161.7 (40-706)	269 ± 184.2 (46-849)	84.6 ± 82.1 (4-481)	3.1 ± 6.1 (0-36)
Median [IQR]	185 [107, 289]	216 [145, 347]	61 [36, 112]	1 [0, 3]

MN: Melanocytic nevi; IQR: Inter-Quartile Range; SD: Standard Deviation.
